# Differential protein expression of Caco-2 cells treated with selenium nanoparticles compared with sodium selenite and selenomethionine

**DOI:** 10.1186/1556-276X-9-589

**Published:** 2014-10-28

**Authors:** Linglin Fu, Xuxia Yan, Xinming Ruan, Junda Lin, Yanbo Wang

**Affiliations:** 1 Key Laboratory for Food Microbial Technology of Zhejiang Province, Food Quality and Safety Department of Zhejiang Gongshang University, 18 Xuezheng Road, Xiasha University Town, Hangzhou 310018, China; 2 College of Life Sciences, Zhejiang University of Traditional Chinese Medicine, 548, Binwen Road, Binjiang District, Hangzhou 310053, China; 3 Department of Biological Sciences, Florida Institute of Technology, 150 W. University Blvd., Melbourne, FL 32901, USA

**Keywords:** Sodium selenite, Selenomethionine, Selenium nanoparticle, Proteomics

## Abstract

The study was designed to determine the differential protein expression of Caco-2 cells treated with different forms of selenium including sodium selenite, selenomethionine (Se-Met), and selenium nanoparticles (nano-Se). Two-dimensional polyacrylamide gel electrophoresis (2D-PAGE) and mass spectrometry (MS) were used to identify the differentially expressed proteins. The results indicated that seven protein spots, ubiquitin-conjugating enzyme E2 (E2), glutathione synthetases (GS), triosephosphate isomerase (TSP), T-complex protein 1 subunit zeta (TCPZ), lamin-B1, heterogeneous nuclear ribonucleoprotein F (hnRNP F), and superoxide dismutase [Cu-Zn] (Cu, Zn-SOD) were significantly different among all the groups. According to the order of control, sodium selenite, Se-Met, and Nano-Se, the expression levels of two proteins (E2 and GS) increased and the other differential proteins were reverse. Except for E2, there were no significant differences in other protein expressions between the groups treated with nano-Se and Se-Met.

## Background

Selenium (Se) is an essential micronutrient for human. Many animal experiments and clinical studies had indicated that Se plays an important role in diverse physiologic actions [1,2]. The essentiality of Se depends on its role at the catalytic site of multiple selenoproteins [3]. Glutathione peroxidase (GSH-Px), thioredoxin reductase (TRR), and deiodinases (ID) are also some of the enzymes with Se in their structure. These enzymes perform many important biological functions. The reduction of hydroperoxide and hydrogen peroxide is catalyzed by GSH-Px through the reduced glutathione. As for TRR, it catalyzes the NADPH-dependent reduction of the redox protein thioredoxin [4]. ID regulates thyroid hormone bioavailability. The above enzymes play a critical role in reproduction, antioxidation, muscle function, and tumor prevention [5].

Although Se is an essential element, its margin between the lowest acceptable level of intake and toxicity is very narrow. According to a previous study, sodium selenite stimulated Caco-2 cell proliferation at low concentrations (10 to 100 nM) but inhibited growth at higher doses (>1 μM) in serum-free medium [6]. The toxicity and bioavailability of Se depend on the chemical form, physiological state, different absorption characteristics, and metabolic pathways [7,8]. In nature and organisms, Se presents as organic (such as selenomethionine (Se-Met) and selenocysteine (Sec)) and inorganic forms (selenide (Se^2-^), selenate (SeO_4_^2-^), selenite (SeO_3_^2-^), and Se) [9-14]. Selenium nanoparticles (nano-Se) is referred to as red or elemental Se, existing as nano-sized particles which usually between 20 and 100 nm [15]. It has been reported previously that nano-Se has high biological activities and low toxicity [16] when compared with other forms of Se. As one of the nanoparticles, nano-Se would have some special physiochemical and biological properties [17]. Therefore, the differences of bioactivity and metabolism of different forms of Se deserve investigation.

It is well known that the small intestine is the main region for absorption of Se [18] and thus it becomes the target organ for Se research. In the intestine of mice, the Sec-containing metabolite is produced by the reaction between Sec and GSH after oral administration of Sec [19]. The Caco-2 cell, differentiated human colon adenocarcinoma cell line, is usually used to mimic human intestine as the cell model. This cell is considered to present morphological and enzymatic characteristics of small intestinal cells and has been generally well accepted for estimating human absorption of various drug and nutrient compounds [20].

In the present study, the Caco-2 cell model, treated with Se in different chemical forms, was established to investigate the corresponding differentially expressed proteins using basal proteomics methods including two-dimensional polyacrylamide gel electrophoresis (2D-PAGE) and mass spectrometry (MS). Therefore, the same concentration of Se at 100 nmol L^-1^ were used in the present study to evaluate and determine the differential protein expressions in the Caco-2 cells treated with sodium selenite, Se-Met, and nano-Se.

## Methods

### Preparation of nano-Se

Nano-Se was prepared according to the previous method described by Zhang et al. [21] through the addition of bovine serum albumin (BSA) to the redox system of selenite and glutathione (GSH). In the present study, 2-mg BSA was dissolved in 4-ml 25-mM GSH and then 1-ml 25-mM sodium selenite was added. The pH value was adjusted to 7.2 using sodium hydroxide. The sizes of red elemental Se was determined using the Mastersizer particle size and zeta potential analyzer (Malvern Instruments, Malvern, UK), with the average sizes being 22.19 nm as nano-Se.

### Cell culture

The cells of Caco-2 were purchased from the Institute of Biochemistry and Cell Biology, Chinese Academy of Science (Shanghai, China). The cells were cultured in Dulbecco's modified Eagle's medium (DMEM) with 10% fetal bovine serum (FBS), 1% nonessential amino acids (NEAA), 2 mM L-glutamine, 100 U ml^-1^ penicillin, and 100 μg ml^-1^ streptomycin at 37°C in a humidified atmosphere of 5% CO_2_ in air. When the cells approximately covered all the bottom of the tissue culture flask, Hanks buffered salt solution (HBSS, pH7.4) was used to wash the cells three times. Then trypsin digestion followed to detach the cells from the culture flask. The reseeded density was 30,000 cells per cm^2^.

### Se exposure and protein extraction

In order to investigate the effects of Se in different chemical forms on the protein expression to Caco-2 cells, sodium selenite, Se-Met, and nano-Se were prepared at 100 nmol L^-1^. Before Se exposure, three forms of selenium sources were all diluted to the required concentration by HBSS. After washing, the Caco-2 cells three times by HBSS, the cells were incubated with HBSS containing 100 nmol L^-1^ of Se in different forms (or only HBSS as control) for 2 h at 37°C in cell incubator. Two hours later, the cells were washed with HBSS triple and collected into 1.5 ml Eppendorf tubes. Then, the electrophoresis cell lysis solution was added to the Eppendorf tube, and the Eppendorf tubes were oscillated about 1 h to ensure the lysis sufficiently. Then, the ultracentrifuge was used to centrifuge the solution for 1 h at 4°C, 40,000 × *g*, and the liquid supernatant was collected and stored at -80°C. Protein quantification was performed using the commercial protein assay kit with BSA as the standard (Jiancheng Biotechnology Co., Nanjing, China).

### Two-dimensional electrophoresis (2DE)

Isoelectric focusing (IEF) was performed on each ReadyStrip IPG strip (17 cm, pH 4 to 7, linear, Bio-rad, Hercules, CA, USA) with IPGphor IEF system (Bio-Rad, USA). Samples containing 100 μg protein were made up to 300 μl with rehydrating buffer (8 M urea, 2 M thiourea, 4% CHAPS, 65 mM dithiothreitol (DTT), 0.33% carrier ampholyte (pH 4 to 6 and pH 5 to 7, respectively)). After the active rehydration for 12 h, IEF was conducted at 250 V for 1 h, 500 V for 1 h, 1,000 V for 1.5 h, 5,000 V for 2 h, 8,000 V for 0.5 h, and 8,000 V to reach a total of approximately 70kVh. After IEF, the strips were equilibrated first, in equilibration buffer I (1% DL-DTT, 50 mM Tris-HCl (pH 6.8), 6 M urea, 30% glycerol, and 2% sodium dodecyl sulfate (SDS)) for 15 min and second, in equilibration buffer II (2.5% iodoacetamide instead of 1% dithiothreitol in equilibration buffer I) for another 15 min. Finally, the second dimension was performed using 12% acrylamide gels, and the 2D-PAGE gels were stained with silver stain method as described by Shevchenko et al. [22].

### Image analysis and protein identification

The gels of 2DE were recorded as digitalized images using a Calibrated Densitometer (GS-800, Bio-Rad, USA), and the search for differentially expressed proteins was carried out using the software of PDQuest Advanced 8.0.1. Protein spots were obtained from the silver-stained gels, detained, and subjected to tryptic digestion as described by Gokulakannan and Niehaus [23] for MS. The peptides mixtures were re-dissolved in matrix solution, dried, and analyzed by a 4800 matrix assisted laser desorption/ionization time of flight mass spectrometry (MALDI-TOF/TOF)(Applied Biosystems, Carlsbad, CA, USA). Combined MS and MS/MS spectra were subjected to MASCOT (version 2.1, Matrix Science, London, UK) by the GPS Explorer software (version 3.6, Applied Biosystems) and searched with the following parameters, National Center for Biotechnology Information Non-redundant (NCBInr) and EST databases. Known contaminant ions (tryptic auto-digest peptides) were excluded. The individual MS/MS spectrum, with a statistically significant (confidence interval ≥95%) ion scores (based on MS/MS spectra), was accepted.

## Results

### Differential proteins analysis

**Figure 1:**
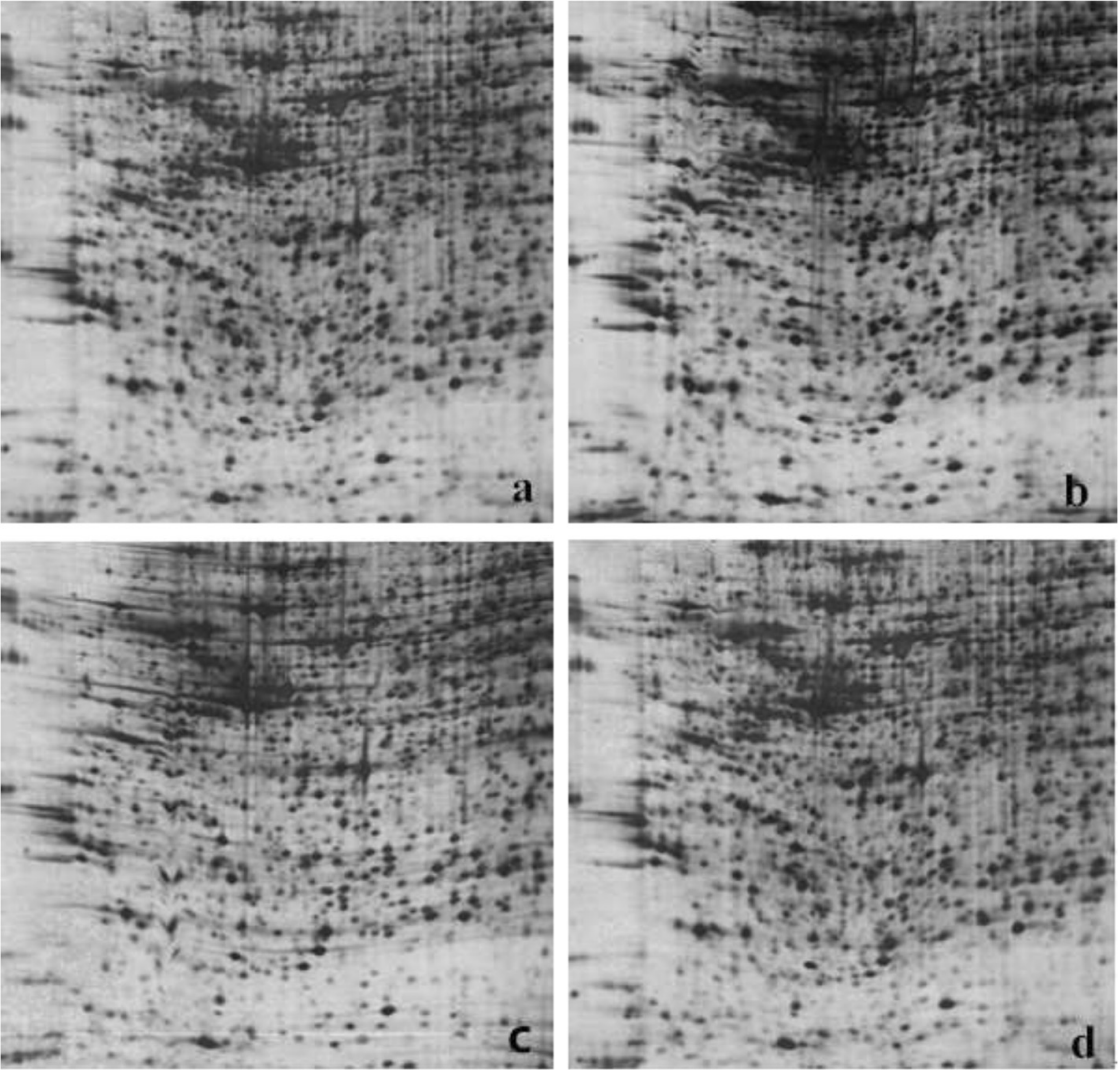
**The two-dimensional electrophoresis (2DE) protein patterns of Caco-2 cells treated with Se. (a)** Control, **(b)** sodium selenite (Na_2_SeO_3_),** (c)** selenomethionine (Se-Met), and **(d)** selenium nanoparticles (nano-Se).

**Figure 2:**
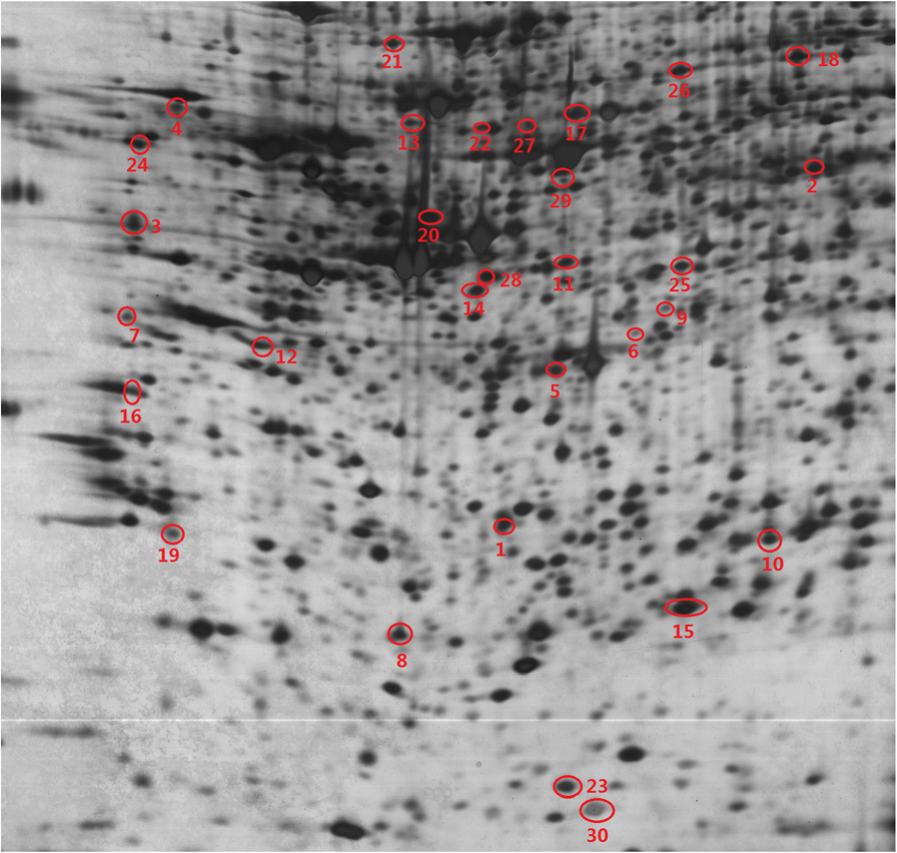
**Representative proteins silver stained gel images of Caco-2 cells.** Seventeen centimeters pH 4 to 7 IPG and 12% SDS-PAGE, SDS-PAGE, sodium dodecyl sulfate polyacrylamide gel electrophoresis. Thirty differential expressed protein spots were marked by red circle.

The whole proteins of Caco-2 cells treated with/without sodium selenite, Se-Met, and nano-Se at 100 nmol L^-1^ were determined by 2D-PAGE. Approximately 700 spots in each gel were detected, and most of the protein spots are similar in the four kinds of 2D-PAGE gels (Figure 1). Differentially expressed protein spots among the four groups of Caco-2 cells were analyzed by PDQuest Advanced 8.0.1 software. As the results, thirty spots represented reproducible upregulated (ratio_(Treatments/Control)_ ≥2, *P* < 0.05) or down-regulated (ratio_(Treatments/Control)_ ≤0.5, *P* < 0.05) changes in different treatments groups compared with those of the control groups (Figure 2).

### Differential proteins identification

**Figure 3:**
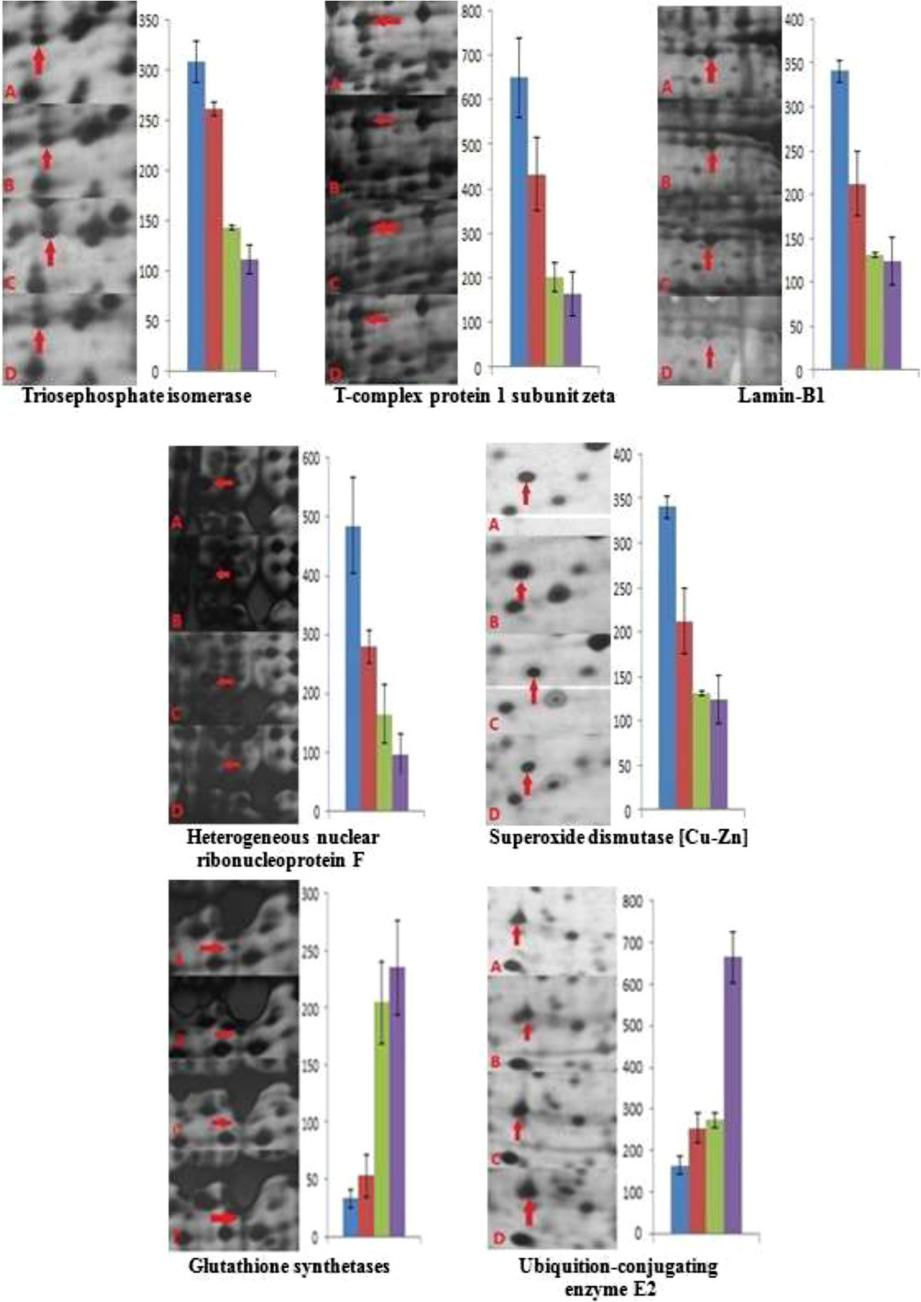
**Enlarged regions and spots densities of 2D gels representative proteins of Caco-2 cells.** From left to right: control, sodium selenite, selenomethionine (Se-Met), and selenium nanoparticles (nano-Se), respectively.

**Figure 4:**
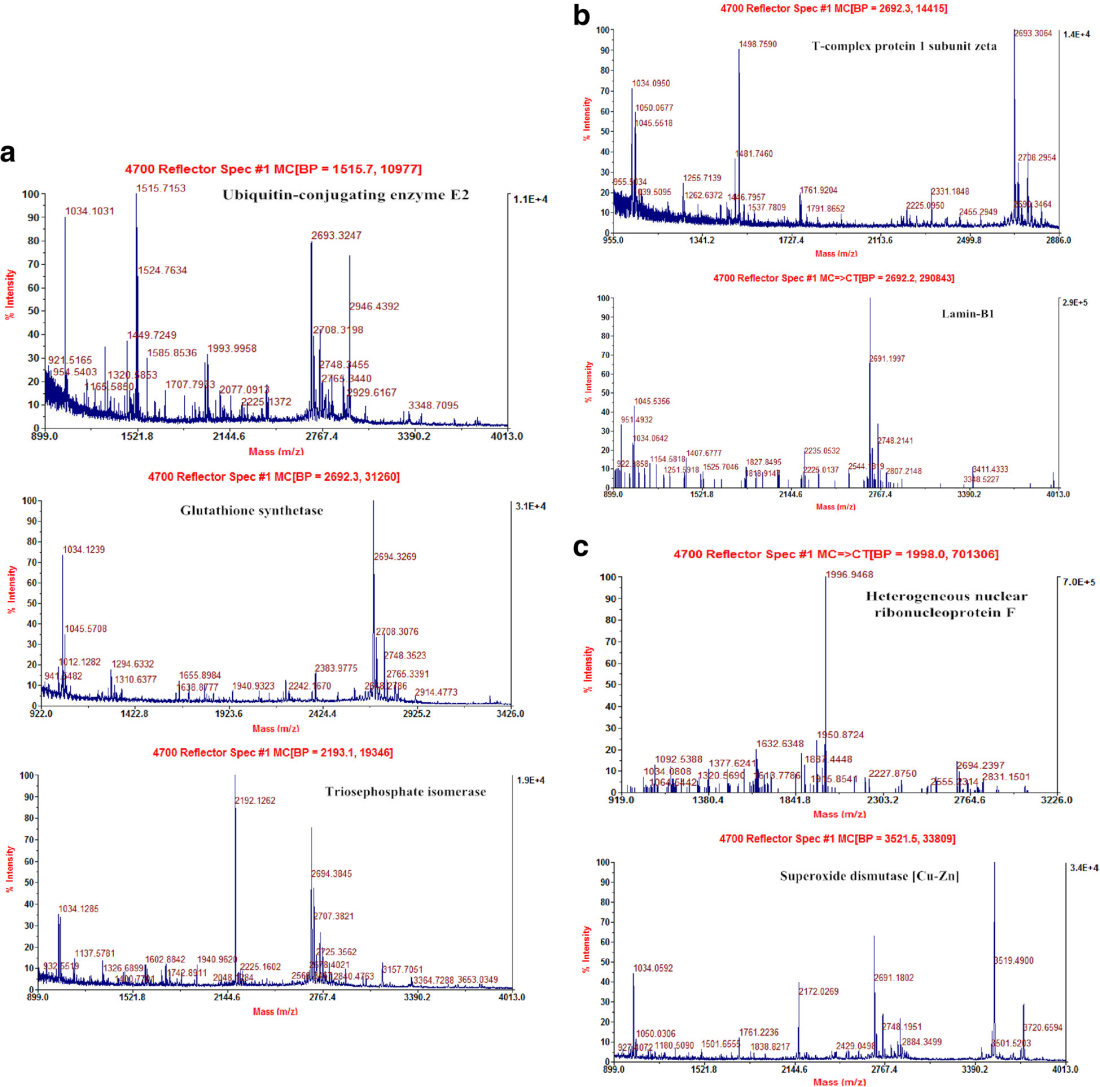
**Peptide mass fingerprinting (PMF) of differential protein spots (a-c).** Proteins including ubiquitin-conjugating enzyme E2 (E2), glutathione synthetases (GS), triosephosphate isomerase (TSP), T-complex protein 1 subunit zeta (TCPZ), lamin-B1, heterogeneous nuclear ribonucleoprotein F (hnRNP F), and superoxide dismutase [Cu-Zn] (Cu, Zn-SOD) were determined using matrix assisted laser desorption/ionization time of flight mass spectrometry (MALDI-TOF-MS).

In order to identify the differentially expressed proteins of Caco-2 cells exposed in different forms of Se with the same concentration, 30 protein spots (marked on Figure 2) were obtained from their corresponding 2-DE gels. After in-gel trypsin digestion and MALDI-TOF/TOF identification, seven significant protein spots were successfully identified as ubiquitin-conjugating enzyme E2 (E2), glutathione synthetases (GS), triosephosphate isomerase (TSP), T-complex protein 1 subunit zeta (TCPZ), lamin-B1, heterogeneous nuclear ribonucleoprotein F (hnRNP F), and superoxide dismutase [Cu-Zn] (Cu, Zn-SOD) (Table 1). The corresponding numbers of the above seven identified protein spots were 8, 29, 10, 18, 21, 20, and 23, respectively (Figure 2). The enlarged regions of each protein in silver stained 2-D gels and the spot density of each protein in each treatment and control group are shown in Figure 3. The MS figures for these seven proteins are listed in Figure 4. According to the order of control, sodium selenite, Se-Met, and nano-Se, the expression levels of two proteins (E2 and GS) increased. As for other proteins expression levels, the results were reversed (Figure 4a,b,c). At the same time, except for E2, there were no significant differences in other protein expressions between the groups treated with nano-Se and Se-Met.

**Table 1:** Differential expressed proteins identified by MALDI-TOF-MS among the four groups of Caco-2 cells

**Accession number**	**Protein name**	**Protein MW**	**Protein PI**	**Protein score (C.I. %)**
sp|P61086|UBE2K_HUMAN	Ubiquitin-conjugating enzyme E2	22,392.6	5.33	97.79
sp|P48637|GSHB_HUMAN	Glutathione synthetase	52,352.3	5.67	95.54
sp|P60174|TPIS_HUMAN	Triosephosphate isomerase	30,771.7	5.65	98.91
sp|P40227|TCPZ_HUMAN	T-complex protein 1 subunit zeta	57,987.6	6.23	95.65
sp|P20700|LMNB1_HUMAN	Lamin-B1	66,367.6	5.11	99.43
sp|P52597|HNRPF_HUMAN	Heterogeneous nuclear ribonucleoprotein F	45,642.9	5.38	99.95
sp|P00441|SODC_HUMAN	Superoxide dismutase [Cu-Zn]	15,925.9	5.7	96.30

## Discussion

Ubiquitin-conjugating enzymes (E2) intervene the ubiquitination and turnover of specific substrates of the ubiquitin-dependent degradation pathway [24]. A conserved core sequence composed of about 150 amino acids from the -NH_2_ terminus consisted in E2 proteins, and cysteine residue is necessary in this region, which plays an essential role in the formation of thiol ester bond with the -COOH terminus of ubiquition [25]. In this study, the expressions of E2 proteins in Caco-2 cells treated with Se were all higher than that of the control groups, and the highest expression of E2 proteins was obtained in the nano-Se groups. Higher expression of E2 proteins indicated the increase of cysteine. Compared with the groups fed with adequate or supranutritional selenium, the significantly decreased levels of plasma homocysteine and cysteine were found in the rats and/or mice fed with selenium-deficient diet [26]. The homocysteine can be converted to cystathionine and cysteine, formed from cystathionine, can be incorporated into protein [26]. Therefore, the three forms of Se may accelerate cysteine increase in the Caco-2 cells and then promote the expression of E2 proteins, and nano-Se has the optimum efficiency to increase cysteine and the expression of E2 protein.

GS catalyzes the ATP-dependent synthesis of glutathione (γ-glutamyl- cysteinyl- glycine; GSH) from γ-glutamyl-cysteine(γ-GC) and glycine. And γ-GC is an intermediate which is produced by γ-glutamyl-cysteine synthetase (GCS) from the amino acid substrates glutamate and cysteine [27]. As described in the E2 protein, Se can accelerate the amino acid substrates cysteine increase, so the activities of GCS could be stimulated subsequently. This hypothesis was verified in the rat liver, and the results indicated that the increase mediated by Se in the activities of GCS was observed [28]. In the present study, the expressions of GS in the groups treated with different forms of Se were all significantly higher than those of the control groups. In addition, the GS in the groups treated with nano-Se and Se-Met were also significantly higher than those treated with sodium selenite. However, the levels of GS were similar and there was no significant difference between groups of nano-Se and Se-Met. All the different forms of Se may increase the activities of GCS in Caco-2 cells, and nano-Se and Se-Met are more effective than others. The increased production of γ-GC should be attributed to the enhancement of GCS activities and then the expressions of GS were stimulated to adapt the demand of synthesis of GSH from γ-GC and glycine.

TSP isomerase catalyzes the reversible interconversion of G3P (glycerol-3-phosphate) and DHAP (dihydroxy acetone phosphate). TSP isomerase is essential for energy production, because only G3P can be used in glycolysis. TSP isomerase allows two molecules of G3P to be produced for every glucose molecule, hence doubling the energy yield. In Se-enriched yeast (SEY), the level of TSP isomerase is lower than that found in regular yeast (RY) [29]. In the present study, the levels of TSP isomerase in groups treated with nano-Se and Se-Met were lower than those treated with sodium selenite and without Se (control). As for the level of TSP isomerase in SEY, it can be concluded that the Caco-2 cells can assimilate more nano-Se and Se-Met than sodium selenite at the same concentration. Nano-Se treatment had been demonstrated to present a higher accumulation of Se compared with sodium selenite treatment in the medaka (*Oryzias latipes*) [30] with the similar exposure way of the present study. Therefore, in this study, nano-Se and Se-Met are more effective in absorption by Caco-2 cell than sodium selenite, with the results that TSP isomerase performed down regulation.

TCPZ is the ζ subunit of T-complex protein 1 (TCP1), known as chaperonin-containing T-complex polypeptide 1 (CCT) or TCP1 ring complex [31]. Regardless of cell type, the expression level of CCT depends on cell growth rate. The upregulation of CCT is to meet the increased requirement for assistance in the folding of proteins which are actively synthesized during cell growth [32]. HnRNP-F is one of the constituents of the splicing-related hnRNP complex and is related to the pre-mRNA cleavage reaction in the mammalian nucleus. And it seems that hnRNP-F might also be related to the cells growth [33]. Different forms of Se exposure induced the different expressed levels of TCPZ and hnRNP-F in Caco-2 cells. Compared with the control groups, significantly more downregulation was found in TCPZ and hnRNP-F of the groups treated with Se. However, no significant differences were observed in TCPZ and hnRNP-F between groups treated with nano-Se and Se-Met. The results indicate that nano-Se has the similar impact with Se-Met to regulate the growth of Caco-2 cells.

The SOD, as a key enzyme in the production of reactive oxygen species (ROS) in cells, catalyzes the dismutation of superoxide anion to oxygen and hydrogen peroxide [34]. In chicks, Cu, Zn-SOD activity was at the higher level if the diet Se was deficiency compared with the normal diet. Se and the increased activity of SOD could be linked with the H_2_O_2_ accumulation [35]. In the present study, the expressions of Cu, Zn-SOD in three Se treatment groups were lower than that of the control groups. It suggested that the less H_2_O_2_ accumulation in the Se treatment groups is associated with the lower expression of Cu, Zn-SOD. In addition, the similar levels of Cu, Zn-SOD were observed between groups treated with nano-Se and Se-Met.

As for lamins, they are important structural components of the nuclear lamina and play a role in nuclear architecture, DNA replication, and gene expression. The lamin-B1 mutant mice provide evidence for a broad and nonredundant function of lamin-B1 in mammalian development [36]. In the present study, the downregulated lamin-B1 was observed in Caco-2 cells treated with Se. In addition, nano-Se and Se-Met also had the similar levels of lamin-B1, and there was no significant difference. It might be related to the metabolic pathway of these two forms Se in Caco-2 cells and the interaction between both nano-Se and Se-Met and lamin-B1 was similar.

## Conclusions

In conclusion, the differentially expressed proteins were determined in Caco-2 cells treated with/without Se (sodium selenite, Se-Met, and nano-Se) using the methods of 2D-PAGE and MS. Thirty spots represented reproducible upregulated or downregulated changes in different Se treatment groups were compared with those of the control, and finally, seven proteins were identified as E2, GS, TSP isomerase, TCPZ, lamin-B1, hnRNP F. and Cu, Zn-SOD. The present study provides the foundation for further understanding the mechanism of proteins associated with Se in Caco-2 cells. In addition, different results were observed in the groups treated with nano-Se, a novel form of Se particle, and thus the reasons and related mechanisms should be investigated in the future study.

## Competing interests

The authors declare that they have no competing interest.

## Authors’ contributions

LF carried out the cell culture, image analysis, and protein identification studies and drafted the manuscript. XY carried out the preparation of nano-Se and the study of two-dimensional electrophoresis and participated in the manuscript preparation. XR carried out the Se exposure and protein extraction study and performed the statistical analysis. YW conceived the study and participated in the design of the study. JL coordinated and helped to draft the manuscript. All authors read and approved the final manuscript.

## References

[B1] Mutakin Meiliana A Wijaya A Kobayashi K Yamazaki C Kameo S Nakazawa M Koyama H Association between selenium nutritional status and metabolic risk factors in men with visceral obesity J Trace Elem Med Biol 2013 27 112 116 10.1016/j.jtemb.2012.09.006 23199701

[B2] Harisa GI Abo-Salem OM El-sayed EM Shazly G Effects of nutritional and excessive levels of selenium on red blood cells of rats fed a high cholesterol diet Biol Trace Elem Res 2013 152 41 49 10.1007/s12011-012-9588-1 23292318

[B3] Stadtman TC Selenocysteine Annu Rev Biochem 1996 65 83 100 10.1146/annurev.bi.65.070196.000503 8811175

[B4] Yan L Johnson LK Selenium bioavailability from naturally produced high-selenium peas and oats in selenium-deficient rats J Agric Food Chem 2011 59 6305 6311 10.1021/jf201053s 21553810

[B5] Mehdi Y Hornick JL Istasse L Dufrasne I Selenium in the environment, metabolism and involvement in body functions Molecules 2013 18 3292 3311 10.3390/molecules18033292 23486107 PMC6270138

[B6] Nano JL Czerucka D Menguy F Rampal P Effect of selenium on the growth of three human colon cancer cell lines Biol Trace Elem Res 1989 20 31 43 10.1007/BF02919096 2484400

[B7] Zeng H Selenium as an essential micronutrient: roles in cell cycle and apoptosis Molecules 2009 14 1263 1278 10.3390/molecules14031263 19325522 PMC6253990

[B8] Xun W Shi L Yue W Zhang C Ren Y Liu Q Effect of high-dose nano-selenium and selenium–yeast on feed digestibility, rumen fermentation, and purine derivative s in sheep Biol Trace Elem Res 2012 150 130 136 10.1007/s12011-012-9452-3 22692882

[B9] Thiry C Schneider YJ Pussemier L Temmerman LD Ruttens A Selenium bioaccessibility and bioavailability in Se-enriched food supplements Biol Trace Elem Res 2013 152 152 160 10.1007/s12011-013-9604-0 23397356

[B10] Algotar AM Stratton MS Ahmann FR Ranger-Moore J Nagle RB Thompson PA Slate E Hsu CH Dalkin BL Sindhwani P Holmes MA Tuckey JA Graham DL Parnes HL Clark LC Stratton SP Phase 3 clinical trial investigating the effect of selenium supplementation in men at high-risk for prostate cancer Prostate 2013 73 328 335 10.1002/pros.22573 22887343 PMC4086804

[B11] Hoffmann PR Berry MJ Seleno protein synthesis: a unique translational mechanism used by a diverse family of proteins Thyroid 2005 15 769 775 10.1089/thy.2005.15.769 16131320

[B12] Hawkes WC Wilhelmsen EC Tappel AL Abundance and tissue distribution of selenocysteine containing proteins in the rat J Inorg Biochem 1985 23 77 92 10.1016/0162-0134(85)83011-7 3156209

[B13] Lamberti C Mangiapane E Pessione A Mazzoli R Giunta R Pessione E Proteomic characterization of a selenium-metabolizing probiotic *Lactobacillus reuteri* Lb2 BM for nutraceutical applications Proteomics 2011 11 2212 2221 10.1002/pmic.201000747 21548091

[B14] Wingler K Bocher M Flohe L Kollmus H Brigelius-Flohe R mRNA stability and selenocysteine insertion sequence efficiency rank gastrointestinal glutathione peroxidase high in the hierarchy of selenoproteins Eur J Biochem 1999 259 149 157 10.1046/j.1432-1327.1999.00012.x 9914487

[B15] Benko I Nagy G Tanczos B Ungvari E Sztrik A Eszenyi P Prokisch J Banfalvi G Subacute toxicity of nano-selenium compared to other selenium species in mice Environ Toxicol Chem 2012 31 2812 2820 10.1002/etc.1995 22927138

[B16] Gao X Zhang J Zhang L Acute toxicity and bioavailability of nano red elemental selenium J Hyg Res 2000 29 57 58 12725047

[B17] Prasad KS Patel H Patel T Patel K Selvaraj K Biosynthesis of Se nanoparticles and its effect on UV-induced DNA damage Colloids Surf B Biointerfaces 2013 103 261 266 23201746 10.1016/j.colsurfb.2012.10.029

[B18] Scharrer E Senn E Wolffram S Stimulation of mucosal uptake of selenium from selenite by some thiols at various sites of rat intestine Biol Trace Elem Res 1992 33 109 120 10.1007/BF02783999 1379448

[B19] Hasegawa T Mihara M Okuno T Nakamuro K Sayato Y Chemical form of selenium containing metabolite in small intestine and liver of mice following orally administered selenocystine Arch Toxicol 1995 69 312 317 10.1007/s002040050176 7654135

[B20] Gammelgaard B Rasmussen LH Gabel-Jensen C Steffansen B Estimating intestinal absorption of inorganic and organic selenium compounds by *in vitro* flux and biotransformation studies in Caco-2 cells and ICP-MS detection Biol Trace Elem Res 2012 145 248 256 10.1007/s12011-011-9174-y 21863324

[B21] Zhang J Wang H Bao Y Zhang L Nano red elemental selenium has no size effect in the induction of seleno-enzymes in both cultured cells and mice Life Sci 2004 75 237 244 10.1016/j.lfs.2004.02.004 15120575

[B22] Shevchenko A Wilm M Vorm O Mann M Mass spectrometric sequencing of proteins from silver-stained polyacrylamide gels Anal Chem 1996 68 850 858 10.1021/ac950914h 8779443

[B23] Gokulakannan GG Niehaus K Characterization of the Medicago truncatula cell wall proteome in cell suspension culture upon elicitation and suppression of plant defense J Plant Physiol 2010 167 1533 1541 10.1016/j.jplph.2010.06.023 20801546

[B24] Haldeman MT Xia G Kasperek EM Pickart CM Structure and function of ubiquitin conjugating enzyme E2-25 K: the tail is a core-dependent activity element Biochemistry 1997 36 10526 10537 10.1021/bi970750u 9265633

[B25] Cook WJ Jeffrey LC Sullivan ML Vierstra RD Three-dimensional structure of a ubiquitin-conjugating enzyme (E2) J Biol Chem 1992 267 15116 15121 1321826 10.2210/pdb1aak/pdb

[B26] Uthus EO Ross SA Dietary selenium affects homocysteine metabolism differently in fisher-344 rats and CD-1 mice J Nutr 2007 137 1132 1136 17449570 10.1093/jn/137.5.1132

[B27] Choi J Opalenik SR Wu W Thompson JA Forman HJ Modulation of glutathione synthetic enzymes by acidic fibroblast growth factor Arch Biochem Biophys 2000 375 201 209 10.1006/abbi.1999.1677 10683268

[B28] Chung AS Maines MD Effect of selenium on glutathione metabolism: induction of γ-glutamylcysteine synthetase and glutathione reductase in the rat liver Biochem Pharmacol 1981 30 3217 3223 10.1016/0006-2952(81)90521-9 6119089

[B29] El-Bayoumy K Das A Russell S Wolfe S Jordan R Renganathan K Loughran TP Somiari R The effect of selenium enrichment on baker's yeast proteome J Proteomic 2012 75 1018 1030 10.1016/j.jprot.2011.10.013 PMC3246083 22067702

[B30] Li H Zhang J Wang T Luo W Zhou Q Jiang G Elemental selenium particles at nano-size (nano-Se) are more toxic to Medaka (*Oryzias latipes*) as a consequence of hyper accumulation of selenium: a comparison with sodium selenite Aquat Toxicol 2008 89 251 256 10.1016/j.aquatox.2008.07.008 18768225

[B31] Yue F Wang L Xia L Wang X Feng B Lu A Chen G Zheng M Modulated T-complex protein 1ζ and peptidyl-prolyl cis-transisomerase B are two novel indicators for evaluating lymph node metastasis in colorectal cancer: evidence from proteomics and bioinformatics Proteomics Clin 2009 3 1225 1235 10.1002/prca.200900028 21136946

[B32] Yokota S Yamamoto Y Shimizu K Momoi H Kamikawa T Yamaoka Y Yanagi H Yura T Kubota H Increased expression of cytosolic chaperonin CCT in human hepatocellular and colonic carcinoma Cell Stress Chaperones 2001 6 345 350 10.1379/1466-1268(2001)006<0345:IEOCCC>2.0.CO;2 11795471 PMC434417

[B33] Balasubramani M Day BM Schoen RE Getzenberg RH Altered expression and localization of creatine kinase B, heterogeneous nuclear ribonucleoprotein F, and high mobility group box 1 protein in the nuclear matrix associated with colon cancer Cancer Res 2006 66 763 769 10.1158/0008-5472.CAN-05-3771 16424007

[B34] Ricardo PU Felipe VP Lucia J Abraham L Cu, Zn superoxide dismutase: cloning and analysis of the *Taenia soliumgene* and *Taenia crassiceps* cDNA Exp Parasitol 2012 130 32 38 10.1016/j.exppara.2011.10.002 22019409

[B35] Avanzo JL Mendonca CX Pugine SMP Cesar MC Effect of vitamin E and selenium on resistance to oxidative stress in chicken superficial pectoralis muscle Comp Biochem Physiol 2001 129(Part C) 163 173 10.1016/s1532-0456(01)00197-1 11423388

[B36] Vergnes L Peterfy M Bergo MO Young SG Reue K Lamin B1 is required for mouse development and nuclear integrity Proc Natl Acad Sci U S A 2004 101 10428 10433 10.1073/pnas.0401424101 15232008 PMC478588

